# Maternal caregiving capabilities are associated with child linear growth in rural Zimbabwe

**DOI:** 10.1111/mcn.13122

**Published:** 2020-12-21

**Authors:** Joice Tome, Mduduzi N. N. Mbuya, Rachel R. Makasi, Robert Ntozini, Andrew J. Prendergast, Katherine L. Dickin, Gretel H. Pelto, Mark A. Constas, Lawrence H. Moulton, Rebecca J. Stoltzfus, Jean H. Humphrey, Cynthia R. Matare, for the Sanitation Hygiene Infant Nutrition Efficacy (SHINE) Trial Team

**Affiliations:** ^1^ Zvitambo Institute for Maternal and Child Health Research Harare Zimbabwe; ^2^ Global Alliance for Improved Nutrition Washington District of Columbia USA; ^3^ Blizard Institute Queen Mary University of London London UK; ^4^ Program in International Nutrition, Division of Nutritional Sciences Cornell University Ithaca New York USA; ^5^ Charles H. Dyson School of Applied Economics and Management Cornell University Ithaca New York USA; ^6^ Department of International Health Johns Hopkins Bloomberg School of Public Health Baltimore Maryland USA

**Keywords:** gender norm attitudes, maternal depression, stunting, women's decision making, women's empowerment, Zimbabwe

## Abstract

Between birth and 2 years, children's well‐being depends on the quality of care they receive from caregivers, primarily their mothers. We developed a quantitative survey instrument to assess seven psychosocial characteristics of women that determine their caregiving ability (‘maternal capabilities’: physical health, mental health, decision‐making autonomy, social support, mothering self‐efficacy, workload and time stress, and gender norm attitudes). We measured maternal capabilities in 4,025 mothers and growth in their 4,073 children participating in the Sanitation Hygiene Infant Nutrition Efficacy (SHINE) trial in rural Zimbabwe. We used generalized estimating equation models with exchangeable correlation structure to test the association between each maternal capability during pregnancy, and infant length‐for‐age *Z* (LAZ) at 18 months, accounting only for within‐cluster correlation and intervention arms in unadjusted analyses and for potential confounders in adjusted analyses to examine the association between each capability, assessed during pregnancy, with child LAZ at 18 months of age. In adjusted models, each unit increase in gender norm attitudes score (reflecting more equitable gender norm attitudes) was associated with +0.09 LAZ (95% CI: 0.02, 0.16) and a decreased odds of stunting (adjusted odds ratio [AOR]: 0.86; 95% CI: 0.74, 1.01); each unit increase in social support score was associated with +0.11 LAZ (95% CI: 0.05, 0.17, *p* < 0.010) and decreased odds of stunting (AOR: 0.83; 95% CI: 0.73, 0.96). Each unit increase in decision‐making autonomy was associated with a 6% reduced odds of stunting (AOR: 0.94; 95% CI: 0.89, 0.996, *p* = 0.04). Interventions and social programming that strengthen these maternal capabilities may improve child nutritional status.

**Key messages**

- The well‐being of young children depends on the quality of care provided by adults—usually their mothers—for feeding, shelter, health care and nurturing stimulation.
- Maternal capabilities are skills and attributes of mothers that determine their ability to translate resources (food, health care, education and shelter) into positive nutrition, health and neurodevelopmental outcomes of their children.
- Among 4,025 rural women in Zimbabwe, holding more equitable gender norm attitudes, having greater decision‐making autonomy and having more social support during pregnancy were each associated with better attained linear growth at 18 months of age in their children.
- Interventions that provide social support to mothers, for example, through home visits or women's groups, may improve health outcome in their children.
- Achieving global child health and nutrition goals will likely require progress toward empowerment of women within their societies and gender equity.

## 1 INTRODUCTION

Among children under 5 years of age, 139 million (22%) are stunted, defined as having a length or height more than 2 standard deviations below the age‐ and sex‐matched international reference population median (UNICEF, World Health Organization [WHO], & World Bank, 2017). Incident stunting is highest between conception and 2 years of age, after which there is little recovery (Shrimpton et al., 2001). Stunting is associated with reduced neurodevelopment and lower educational attainment during childhood (Dewey & Begum, 2011) and reduced economic productivity during adulthood (Hoddinott et al., 2013). Thus, stunting is an indicator of lifelong lost human capital, and interventions to prevent stunting must occur before 2 years of age.

Virtually all interventions to improve linear growth and all other nutrition and health outcomes in infants and young children (e.g. feeding, and provision of hygienic living conditions and health care) rely on implementation by a caregiver. In most contexts, this is primarily the child's mother. The UNICEF Framework on malnutrition highlighted ‘nurturing care’ as a critical requirement for healthy child growth and development (UNICEF, 1990). Differences in the quality of care that children receive likely contribute to heterogeneity in nutritional status among children living in the same community with similar access to resources. Accordingly, we sought to better understand caregiving behaviour by identifying underlying psychosocial characteristics of women that determine caregiving ability. Building on the extended UNICEF model of care (Engle, Menon, & Haddad, 1999), the human capabilities theory (Nussbaum, 2001) and empirical studies, we developed a ‘maternal capabilities’ construct. We defined maternal capabilities as the characteristics of a mother that determine her ability to care for a young child in ways that produce positive nutrition, health and developmental outcomes. Our construct includes seven capabilities: physical and mental health, decision‐making autonomy, social support, mothering self‐efficacy, workload and time stress, and gender norm attitudes. We developed a quantitative tool to measure these maternal capabilities, primarily drawing on previously published instruments (Matare, Mbuya, Pelto, Dickin, & Stoltzfus, 2015). We defined strong maternal capabilities as good physical and mental health, high levels of social support and mothering self‐efficacy, high autonomy for decision making within the household, egalitarian gender norm attitudes and low levels of perceived time stress. We hypothesized that children of mothers with strong maternal capabilities will receive better care and therefore have better nutritional status than children of mothers with weak maternal capabilities.

## 2 METHODS

The newly developed maternal capabilities tool is available at https://osf.io/w93hy; Table 1 summarizes the definition, justification and method of measurement for each maternal capability. In the current study, we administered the newly developed tool to pregnant women enrolled in the Sanitation Hygiene Infant Nutrition Efficacy (SHINE) trial in rural Zimbabwe (SHINE Trial team, 2015). The SHINE trial tested the impact of two interventions (an infant and young child feeding [IYCF] intervention and a water, sanitation and hygiene [WASH] intervention) on child linear growth at 18 months. In the current study, we hypothesized that children of SHINE mothers with strong maternal capabilities would have greater length‐for‐age *Z* (LAZ) and less stunting at 18 months of age.

**TABLE 1:** The maternal capabilities construct implemented in the SHINE trial: Definitions, justifications and measurement methods

Maternal capability	Definition	Justification	Measurement
Decision‐making autonomy	Choice or control in decisions and access to resources that affect the mother herself or her children (Dixon‐Mueller, 2013)	Child nutritional status is generally associated with maternal autonomy, in particular maternal control over health care decision (Carlson, Kordas, & Murray‐Kolb, 2015).	Questions were taken from the Zimbabwe Demographic and Health Survey 2015; questions ask whether a mother can make a decision on her own to purchase 7 items of varying value
Gender norm attitudes	Egalitarian gender norm attitudes are a belief that men and women should have equal access to resources and opportunities	Mothers with more egalitarian attitudes are more likely to have an institutional delivery and a fully immunized child and to practice exclusive breastfeeding (Mbuya et al., 2019; Singh, Haney, & Olorunsaiye, 2013).	Questions adapted from the Gender Norm Attitudes Scale (Nanda, 2011)
Mental health	Depression; loss of interest or pleasure in usual activities, reduced energy, feelings of guilt, low self‐worth, disturbed sleep or appetite, poor concentration	Depression reduces maternal–child interaction (Poobalan et al., 2007); child health‐care seeking (Avan, Richter, Ramchandani, Norris, & Stein, 2010) has a negative impact on caregiving (WHO, 2012) and is associated with poor nutritional status (Surkan, Kennedy, Hurley, & Black, 2011).	The Edinburgh Postnatal Depression Scale was used (Cox, Holden, & Sagovsky, 1987), which has been translated and validated in Shona among Zimbabwean women (Chibanda et al., 2010)
Mothering self‐efficacy	A woman's self‐confidence in her role as a competent mother	Self‐efficacy is a proximal determinant of behaviour in many models (Bandura, 1977; Becker, 1974; Fishbein, 1979). There is a strong evidence that self‐efficacy is an important determinant of breastfeeding (Tuthill, McGrath, Graber, Cusson, & Young, 2016).	Questions adapted from the Parenting Sense of Competence Scale (Dumka, Stoerzinger, Jackson, & Roosa, 1996; Gilmore & Cuskelly, 2009) and the Parenting Self‐Agency Measure
Perceived physical health	The extent to which a woman perceives herself to be free of disease and in good health	Women in low‐ and middle‐income countries face high burdens of disease (Murray et al., 2012), which may reduce their energy levels and quality of childcare (Engle, Menon, & Haddad, 1999).	Adapted from the RAND 36‐Item Health Survey (Hays, Sherbourne, & Mazel, 1993)
Social support	Access to relationships that provide physical, psychological and informational resources, which help the mother feel she belongs, is competent and can cope.	Included in many models of health behaviour (Bronfenbrenner, 1986). Important for breastfeeding behaviours (Haider, Ashworth, Kabir, & Huttly, 2000). The functions of social support are informational, instrumental, emotional and companionship (Cohen, Mermelstein, Kamarck, & Hoberman, 1985); this type of social support, when provided through care group leaders (Hossain, 2020) or by peer counsellors, is associated with child health (Saleem, Mahmud, Baig‐Ansari, & Zaidi, 2014).	Questions adapted from the Interpersonal Support Evaluation List (Cohen, Mermelstein, Kamarck, & Hoberman, 1985) and the Medical Outcomes Study Social Support Survey (Sherbourne & Stewart, 1991)
Time stress	The extent to which a mother feels she does not have adequate time to complete all her responsibilities.	Women's work burden may negatively influence childcare because women's time in low‐ and middle‐income countries is a zero‐sum game: new activities can only be added at the expense of others (Engle, Menon, & Haddad, 1999).	Questions developed for the SHINE trial. Questions ask whether the mother feels stress, unhappiness or worry about her work load

SHINE was a cluster‐randomized community‐based trial conducted in two contiguous rural districts in central Zimbabwe (Chirumanzu and Shurugwi); methods have been previously described (SHINE Trial Team, 2015). Briefly, clusters were defined as the catchment area of one to four village health workers (VHWs) employed by the Ministry of Health and Child Care (MoHCC). The clusters were randomly allocated to one of four treatment groups: Standard of Care (SOC, promotion of uptake of MoHCC maternal–child health services, early breastfeeding initiation and exclusive breastfeeding to 6 months of age), WASH (all SOC interventions plus provision of a latrine, handwashing stations, soap, chlorine for point‐of‐use drinking water treatment and a play space to reduce infant geophagia with promotion of hygiene behaviours); IYCF (all SOC interventions plus 20 g of daily small‐quantity lipid‐nutrient supplement between infant ages 6 to 18 months, and promotion of optimal complementary feeding practices); or WASH+IYCF (all interventions delivered concurrently). From 22 November 2012 to 27 March 2015, VHWs prospectively identified and referred new pregnancies to the trial. Research nurses, employed by the trial, confirmed the pregnancy and enrolled women at a median [IQR] gestational age of 12 [9, 16] weeks, following written informed consent. VHWs made monthly home visits delivering arm‐specific behaviour change interventions and commodities. Research nurses made home visits to assess baseline characteristics of mothers and their households (baseline visit was ~2 weeks after enrolment), infant's characteristics at birth and infant length at 18 months. At baseline, maternal anthropometric measures were obtained, haemoglobin was measured (HemoCue, Angelholm, Sweden), and HIV status was determined via rapid test algorithm. HIV‐positive women were urged to seek immediate antenatal care for prevention of mother‐to‐child transmission. Maternal and household characteristics were elicited using a structured questionnaire. Date of last menstrual period (LMP) was recorded. Household food security status was assessed using the Coping Strategies Index (Maxwell, Watkins, Wheeler, & Collins, 2003) and relative wealth using an asset‐based index as described (Chasekwa et al., 2018). Maternal capabilities were assessed by the maternal capabilities survey. Mothers diagnosed with clinical depression, as measured by the Edinburgh Postnatal Depression Scale (EPDS) score ≥ 12 and/or suicidal ideation (a categorization that has been validated against clinical diagnosis among Zimbabwean women) (Chibanda et al., 2010), were referred for further assessment. Infant birth weight and delivery details were transcribed from health records; 89% of infants were delivered in a health institution (Humphrey et al., 2019); and the trial provided Tanita BD‐590 (Arlington Heights, IL, USA) infant scales to all health institutions in the study area and trained the facility staff on how to use the scales. Gestational age at delivery was calculated from date of LMP. At 18 months, recumbent infant length was measured and recorded three times to the nearest 0.1 cm using a Seca 417 infantometer (Weigh & Measure LLC., Olney, MD, USA); the median value was used in analysis. Research nurses were standardized every 6 months against a gold‐standard anthropometrist.

Primary results of the SHINE trial showed that the IYCF, but not the WASH, intervention improved child linear growth (Humphrey et al., 2019; Prendergast et al., 2019). We also observed a higher prevalence of stunting among HIV‐uninfected children born to HIV‐positive mothers than among children of HIV‐negative mothers (stunting prevalence was 50% compared with 32%). Accordingly, our statistical analysis plan (SAP) prespecified that we would test for effect modification between IYCF treatment and each maternal capability, and between maternal HIV infection and each maternal capability on infant LAZ at 18 months. There is also evidence that mothers care for boys differently than girls (Yount, 2004), so we prespecified testing interaction between infant sex and maternal capabilities on growth. Several studies have reported that maternal depression during pregnancy is associated with reduced birth weight (Field et al., 2004; Nasreen, Kabir, Forsell, & Edhborg, 2010); therefore, we hypothesized that any observed associations between maternal capabilities and LAZ at 18 months could be mediated through birth weight. In summary, we prespecified our plan to test for interaction between the maternal capabilities and three factors (maternal HIV status, infant sex and the IYCF treatment arm) on infant LAZ at 18 months and to determine if any observed associations between maternal capabilities and infant LAZ at 18 months were mediated by birth weight.

### 2.1 Statistical methods

All mother–infant dyads enrolled in SHINE who provided maternal capability information at baseline and child LAZ at 18 months were included in this analysis. LAZ was calculated according to WHO Child Growth Standards; stunting was defined as LAZ < −2.0. The EPDS scores were modelled as depressed (score ≥ 12 and/or suicidal ideation) or not depressed (Chibanda et al., 2010). Decision‐making autonomy was calculated as the sum of five questions, each of which could be scored as 0 (no) or 1 (yes). The median (IQR) decision‐making autonomy score was 5 (4, 5) out of a range of possible scores of 0–5; accordingly, we modelled this variable as a binary variable, where 1 indicated a score of 5 and 0 indicated a score < 5. For each of the other maternal capabilities, the mean of several Likert‐type items was calculated to give a composite score.

#### 2.1.1 Associations between maternal capabilities and child linear growth

In unadjusted analyses, we used generalized estimating equation (GEE) models with exchangeable correlation structure to test the association between each maternal capability during pregnancy, and infant LAZ at 18 months, accounting only for within‐cluster correlation and intervention arms (i.e. IYCF vs. no IYCF and WASH vs. no WASH). In adjusted analyses, we considered covariates that had been identified as common predictors of linear growth in the literature and that were prespecified in a statistics (https://osf.io/w93hy). We used a forward stepwise selection procedure with *p* < 0.2 to enter to arrive at the final set of covariates. Results of GEE analyses were expressed as regression coefficients (for LAZ analyses) or odds ratios (for stunting analyses), with 95% confidence intervals (CIs) around point estimates.

#### 2.1.2 Effect modification

We hypothesized that the association of the maternal capabilities with child LAZ may be modified by three factors: infant sex, maternal HIV status or the IYCF intervention. We used regression analysis to calculate interaction terms. Where the interaction was significant (*p* < 0.05), stratified analyses were conducted. For maternal capabilities that were not binary, we categorized them as high or low at the median.

#### 2.1.3 Effect mediation

We tested the association of each maternal capability with birth weight using the same GEE modelling approach as used for LAZ. If any maternal capability was significantly associated with both birth weight and LAZ, we used a mediator model to investigate whether the association between the capability and LAZ was mediated through birth weight.

All analyses were performed using STATA version 14.1 (College Station, TX: StataCorp, LP).

### 2.2 Ethical considerations

The Medical Research Council of Zimbabwe and the Institutional Review Board of the Johns Hopkins Bloomberg School of Public Health reviewed and approved the study protocol.

## 3 RESULTS

**FIGURE 1:**
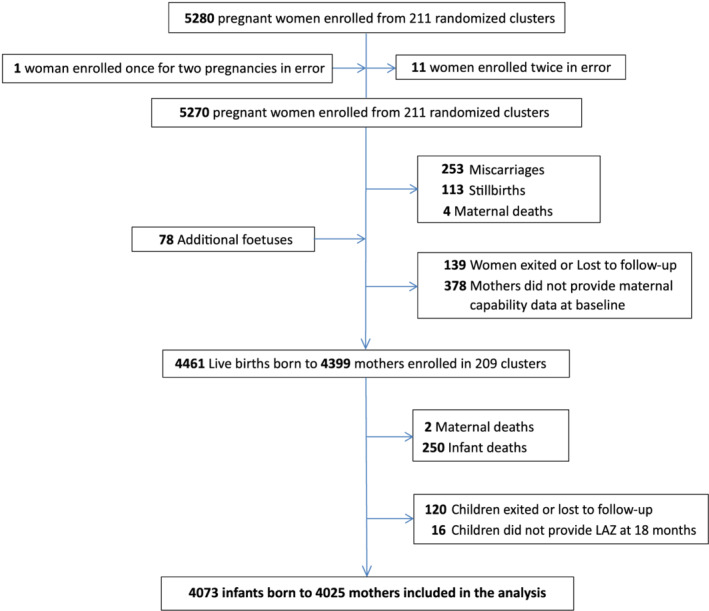
Flow of participants through the study

Among 5,280 women enrolled in SHINE, 11 were excluded and one was added owing to enrolment errors; 370 had miscarriages, had still births or died during pregnancy; 139 were lost to follow‐up or exited from the trial before delivery; and 378 mothers did not provide maternal capability data at baseline (Figure 1). With the addition of 78 fetuses in twin pregnancies, there were 4,461 live births born to 4,399 mothers with maternal capability data. Of these, 250 children and two mothers died before 18 months post‐partum, 120 children exited or were lost to follow‐up, and 16 infants did not provide a LAZ at 18 months, leaving 4,073 live births born to 4,025 mothers included in the current analysis. Compared with mothers who were included in the analysis, mothers who were missing maternal capabilities during pregnancy (exposure variables) or LAZ at 18 months (outcome) were less likely to be married, more likely to have an unknown HIV status and had 5.6 days' shorter gestation; other baseline characteristics were similar (Table S2). At baseline, mothers had a mean (SD) age of 26.3 (6.6) years and were generally well‐nourished with a mean (SD) mid‐upper arm circumference of 26.4 (3.0) cm (Table 1). Most of the women were married. Sixteen per cent of the women were HIV positive. Less than half of the households had a latrine, and about one third obtained drinking water from an unimproved source.

### 3.1 Association of maternal capabilities with child linear growth

At 18 months post‐partum, the mean (SD) LAZ was −1.6 (1.1), and one third of the children were stunted. In univariable analyses, children of mothers with greater decision‐making autonomy, more egalitarian gender norm attitudes, fewer depressive symptoms and higher levels of social support and mothering self‐efficacy had significantly better linear growth outcomes (LAZ and/or stunting) at 18 months (Table 2). In adjusted analyses, gender norm attitudes and social support remained significantly associated with LAZ, and decision‐making autonomy and social support remained significantly associated with stunting. Mothers with greater time stress during pregnancy were also more likely to have a child with lower LAZ at 18 months, although this association did not reach statistical significance (−0.04; 95% CI: −0.08, 0.004, *p* = 0.07). In post hoc analyses, we repeated these analyses with the outcome of LAZ and stunting at 1, 3, 6 and 12 months post‐partum. Mothers who perceived themselves to be in better health compared with worse health had children with better linear growth at 1 and 12 months of age (Tables S4A and S4B). Similar to 18‐month findings, maternal decision‐making autonomy, social support and time stress were associated with child linear growth at 12 months (Table S4B).

**TABLE 2:** Baseline and perinatal characteristics of study participants^a^

*Maternal and household factors at baseline*	*N* = 4,025
Maternal sociodemographic	
Mean age (SD), years	26.3 (6.6)
Mean height (SD), cm	160.2 (5.9)
Mean weight (SD), kg	61.0 (9.8)
HIV status, % (*n*/*N*)	
Positive	16.0 (642/4,025)
Negative	83.8 (3,374/4,025)
Unknown	0.2 (9/4,025)
Mean years of schooling completed (SD)	9.5 (1.8)
Married, % (*n*/*N*)	95.6 (3,660/3,828)
Employed, % (*n*/*N*)	8.7 (348/4,007)
Religion	
Apostolic, % (*n*/*N*)	46.5 (1,797/3,861)
Other Christians, % (*n*/*N*)	45.0 (1,737/3,861)
Other religions, % (*n*/*N*)	8.5 (327/3,861)
Maternal capabilities	
Median decision‐making autonomy score [IQR]	5 [4–5]
Median gender norm attitudes score [IQR]	3 [2.7–3.5]
Median perceived time stress score [IQR]	2.6 [2–3.2]
Median perceived social support score [IQR]	3.6 [3.3–4]
Median perceived health status score [IQR]	3.6 [2.7–4.2]
Median mothering self‐efficacy score [IQR]	4 [3.8–4.2]
Depression, % (*n*/*N*)	8.7 (343/3,941)
Household	
Median number of occupants [IQR]	5 [3–6]
Median number of children [IQR]	2 [1–3]
Median number of children under 5 years [IQR]	1 [0–1]
Any latrine at household, % (*n*/*N*)	40.1 (1,588/3,958)
Household meets minimum diet diversity score, % (*n*/*N*)	34.7 (1,395/4,025)
Median coping strategy index score [IQR]	1 [0–7]
Main source of household drinking water improved, % (*n*/*N*)	62.8 (2,493/3,968)
Improved floor, % (*n*/*N*)	54.2 (2,148/3,963)

Infant characteristics at birth	*N* = 4,073
Mean LAZ (SD)	−1.6 (1.1)
Stunting, % (*n*/*N*)	33.4 (1,361/4,073)
Female sex, % (*n*/*N*)	49.9 (2,031/4,073)
Mean birth weight (SD), *kg*	3.1 (0.5)
Birth weight < 2,500 g, % (*n*/*N*)	8.7 (325/3,740)
Mean gestational age (SD),weeks	38.6 (3.7)
Multiple birth, % (*n*/*N*)	2.7 (108/4,073)

Abbreviations: IQR, interquartile range; LAZ, length‐for‐age *Z*.

^a^ Percentages are calculated from available data for each variable; however, a missing category was included where a variable was >10% missing.

**FIGURE 2:**
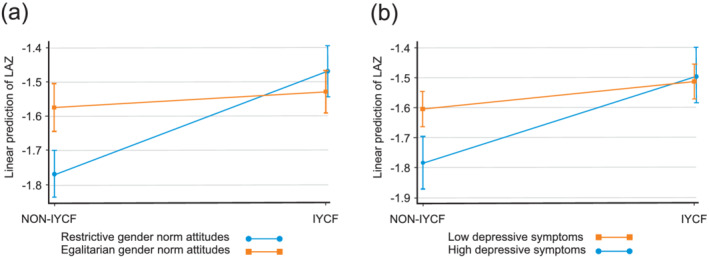
The effect modification on length‐for‐age *Z* (LAZ) outcomes in the Sanitation Hygiene Infant Nutrition Efficacy (SHINE) trial. (A) Effect of infant and young child feeding (IYCF) intervention on LAZ stratified by gender norm attitudes (interaction term, *p* < 0.001). (B) Effect of IYCF intervention on LAZ stratified by depressive symptoms (interaction term, *p* = 0.036). Among children in the non‐IYCF arms, those whose mothers are with restrictive gender norm attitudes and those whose mothers are with depression had lower LAZ than those whose mothers had stronger maternal capabilities. But among children in the IYCF arm, children of mothers with weak maternal capabilities benefited from the IYCF intervention more than those whose mothers are with strong capabilities: The IYCF intervention ameliorated the adverse association of weak maternal capabilities on linear growth

The IYCF intervention significantly modified the associations of depressive symptoms (*p* value of interaction term = 0.029) and gender norm attitudes (*p* value of interaction term < 0.001) with child LAZ at 18 months. Stratified analyses are presented in Table 2. To illustrate these interactions, we plotted the mean LAZ of children who did and did not receive the IYCF intervention stratified by those whose mothers held restrictive and egalitarian gender norm attitudes (Figure 2A) and by those whose mothers were and were not depressed (EPDS during pregnancy; Figure 2B). Among children who did not receive the IYCF intervention, those whose mothers held restrictive compared with egalitarian gender norm attitudes and those whose mothers were depressed compared with not depressed were shorter. However, the LAZ of children who received the IYCF intervention did not differ by their mother's gender norm attitudes or depression. These graphs illustrate that the SHINE IYCF intervention ameliorated the adverse associations of child linear growth with maternal inequitable gender norm attitudes and with maternal depression. There were no other significant interaction terms between a maternal capability assessed during pregnancy and the IYCF intervention or child sex or maternal HIV status.

We found no evidence to support our hypothesis that the association between maternal capabilities assessed during pregnancy and child LAZ at 18 months is mediated through birth weight. Mothering self‐efficacy was associated with birth weight (Table 3), but it was not associated with child LAZ at 18 months (Table 2). None of the other maternal capabilities were associated with birth weight (Table 3).

**TABLE 3:** Association of maternal capabilities assessed during pregnancy with child length‐for‐age *Z* score and stunting at 18 months of age

Maternal capability	Maternal capacity assessed during pregnancy			
	Length‐for‐age *Z* score at 18 months		Stunted (LAZ < −2.0) at 18 months	
	Unadjusted *β* (95% CI)^a^	Adjusted *β* (95% CI)^b^	Unadjusted OR (95% CI)^a^	Adjusted OR (95% CI)^b^
Decision‐making autonomy	0.04 (0.01, 0.06) 0.01	0.01 (−0.02, 0.04) 0.42	0.92 (0.87, 0.96) <0.01	0.94 (0.89, 0.996) 0.04
Gender norm attitudes^c^	0.12 (0.05, 0.18) <0.01	0.09 (0.02, 0.16) 0.02	0.82 (0.72, 0.95) 0.01	0.86 (0.74, 1.01) 0.06
IYCF group	−0.01 (−0.11, 0.08) 0.77	−0.04 (−0.13, 0.05) 0.39	1.02 (0.83, 1.24) 0.86	1.09 (0.88, 1.35) 0.43
Non‐IYCF group	0.24 (0.15, 0.33) <0.01	0.18 (0.08, 0.29) <0.01	0.68 (0.56, 0.82) <0.01	0.68 (0.54, 0.86) <0.01
Depression (EPDS ≥ 12 or suicidal^d^)	−0.11(−0.24, 0.02) 0.10	−0.02(−0.14, 0.11) 0.80	1.27(0.97, 1.65) 0.08	1.10(0.81, 1.49) 0.54
IYCF group	0.03 (−0.13, 0.19) 0.70	0.07 (−09, 0.24) 0.38	1.04 (0.71, 1.53) 0.84	0.96 (0.62, 1.48) 0.84
Non‐IYCF group	−0.24(−0.43, −0.04) 0.02	−0.08 (−0.24, 0.09) 0.37	1.49 (1.03, 2.15) 0.03	1.21 (0.81, 1.79) 0.35
Perceived social support	0.13 (0.07, 0.20) <0.01	0.11 (0.05, 0.17) <0.01	0.79 (0.69, 0.91) <0.01	0.83 (0.73, 0.96) 0.01
Mothering self‐efficacy	0.06 (−0.02, 0.14) 0.14	0.03 (−0.06, 0.11) 0.51	0.86 (0.74, 1.00) 0.05	0.93 (0.78, 1.09) 0.36
Perceived health status	0.01 (−0.03, 0.04) 0.74	0.01 (−0.02, 0.04) 0.57	1.00 (0.93, 1.08) 0.96	1.00 (0.92, 1.09) 0.93
Perceived time stress	−0.03 (−0.07, 0.02) 0.26	−0.04 (−0.08, 0.004) 0.07	1.01 (0.92, 1.10) 0.89	1.01 (0.91, 1.12) 0.80

Abbreviations: EPDS, Edinburgh Postnatal Depression Scale; IYCF, infant and young child feeding; LAZ, length‐for‐age *Z*.

^a^ Unadjusted models—adjusted for cluster and intervention arms to account for within‐cluster correlation and controlling for the intervention effect.

^b^ Adjusted models—adjusted for cluster, intervention arms and other baseline covariates, which were associated with the exposure and outcome at *p* < 0.2. Baseline variables tested: age of mother at birth, mother's height, mother's body mass index, mother's mid‐upper arm circumference, mother's level of education, receipt of water, sanitation and hygiene (WASH) intervention, receipt of IYCF intervention, child birth order, child sex, single or multiple birth, birth interval, time to water source, improved latrine, improved water source, household wealth index, presence of functioning hand washing station with water and soap, coping strategy index, gender of household head, faeces observed in yard, number of children under‐fives, household size, treated water, father's level of education, mother's employment outside home, household dietary diversity and mother's HIV status at enrolment. See Table S3 for lists of covariates retained in each final adjusted model.

^c^ Interaction term between IYCF * Gender norm attitudes was significant (*p* < 0.001), so stratified analyses are presented showing that the association of gender norm attitude with LAZ was apparent only for children who did not receive the IYCF intervention.

^d^ Interaction term between IYCF * Depression was significant (*p* = 0.036), so stratified analyses are present showing that the association of depression with LAZ was apparent for children who did not received the IYCF intervention, although after adjustment, this association was not statistically significant.

## 4 DISCUSSION

In this population of 4,025 mothers in rural Zimbabwe, those who reported greater decision‐making autonomy, more egalitarian gender norm attitudes and higher levels of social support during pregnancy had children who attained better linear growth by 18 months.

Decision‐making autonomy was very high among SHINE women: out of a possible score of 5, the median score during pregnancy was 5 (IQR: 4, 5). Yet even within this tight distribution, each unit increase in score was significantly associated with a 6% reduced odds of having a stunted child. Our findings on gender norms are consistent with a recent cross‐sectional analysis of Demographic and Health Survey data from five east sub‐Saharan African countries that reported that maternal gender norm attitudes (termed ‘intrinsic agency’ in that paper) were significantly associated with child height‐for‐age difference *Z* (HAZ); the effect was both direct and mediated through maternal body mass index, suggesting that holding restrictive gender norm attitudes may negatively affect self‐care as well as childcare (Jones et al., 2019). Our findings on social support suggest that the positive child health effects of programmes that provide child health and nutrition education through regular home visits (e.g. The Care Group strategy, Perry et al., 2015; Lady Health Visitors in Pakistan, Upvall, Sochael, & Gonsalves, 2002; or mothers' support groups, Undlien, Viervoll, & Rostad, 2016) may be mediated through increased levels of social support for mothers. The magnitude of linear growth associated with gender norms and social support scores was substantial. Our results predict that SHINE mothers who had optimal scores for gender norm attitudes and social support (5 for both domains) during pregnancy, as compared with mothers who had the median scores (3.0 [IQR: 2.7–3.5] for gender norm attitudes and 3.6 [IQR: 3.3–4] for social support) had children who were, respectively, 0.18 LAZ and 0.16 LAZ longer at 18 months. This effect size can be compared with that of the IYCF intervention on LAZ among HIV‐unexposed uninfected (HUU) children in SHINE (0.16; 95% CI: 0.08, 0.23) (Humphrey et al., 2019).

Maternal depression during pregnancy has been frequently reported to predict low birth weight (Nasreen, Kabir, Forsell, & Edhborg, 2010; Rahman, Bunn, Lovel, & Creed, 2007; Wado, Afework, & Hindin, 2014). In our study, although not statistically significant, depressed mothers were 35% *less* likely to have a low birth weight baby (OR; 95% CI: 0.65 0.39, 1.09, *p* = 0.10). Our study sample had a relatively low prevalence of antenatal depression (8.8%), compared with studies that have found this association in populations with antenatal depression > 20% (Chang et al., 2014; Wado, Afework, & Hindin, 2014). We have previously demonstrated that maternal depression, gender norm attitudes and social support assessed during pregnancy predicted several childcare behaviours (institutional delivery, breastfeeding initiation, exclusive breastfeeding, having a fully immunized child and complementary feeding) (Matare et al., 2020). In the current analysis, we demonstrate that these same capabilities in pregnancy are not associated with birth weight. Together, these observations suggest that maternal capabilities most likely affect child growth through improved care practices rather than a biologic pathway.

We did not observe associations between linear growth and perceived health status. Notably, most mothers were in good health status (well‐nourished, 80% of HIV+ women were taking antiretroviral therapy (Prendergast et al., 2019) and had very little anaemia (personal communication, JH Humphrey). It may be that some mothers perceived themselves to be in worse health than they were and that their objective health status did not constrain childcare. We also did not observe an association between linear growth and mothering self‐efficacy. This may be because our tool reflected global mothering self‐efficacy; it may be that self‐efficacy in specific skills more directly related to child linear growth (e.g. complementary feeding) may be associated with linear growth.

In the past 5 years, four review papers have summarized the literature on maternal empowerment (defined as women's ability to make strategic life choices) and child nutritional status (Carlson, Kordas, & Murray‐Kolb, 2015; Cunningham et al., 2015; Hossain, 2020; Pratley, 2016; Santoso et al., 2019): all reported that mothers' empowerment was generally associated with better child nutritional status, but with many reported associations not reaching statistical significance and many inconsistencies between studies. A major obstacle to interpreting this literature is the wide variety of definitions and tools used in assessing empowerment. In most studies ‘empowerment’ has included some assessment of decision‐making autonomy (Aslam & Kingdon, 2012; Desai & Jain, 1994; Hossain, 2020; Shroff, Griffiths, Adair, Suchindran, & Bentley, 2009); some studies have included women's mobility (Sethuraman, Lansdown, & Sullivan, 2006), and others have relied on nonspecific indicators like education, employment and household size (Shafiq et al., 2019). In developing the set of tools used in the current study, we took a broader approach to assess a comprehensive set of skills and attributes that aimed to distinguish women who are able to take optimal advantage of available resources to care for their children from those with similar resources who are less capable. In our construct, the ability to make strategic life choices is just one determinant: a woman's physical and mental health, her self‐confidence in being a mother and her role in society are also critical. Future research should test these tools in other contexts and elucidate the mechanisms linking capabilities to child health outcomes.

Our study has two important limitations. First, although the longitudinal design provides stronger evidence for evaluating the relationship between early exposures and child health outcomes than cross‐sectional studies, the data are observational, subject to confounding by unmeasured factors in drawing causal inference. Our study has identified specific maternal factors that may particularly influence child health outcomes; future studies could test the impact of interventions to develop or strengthen these skill and qualities in mothers using a randomized design. Second, of the seven survey tools developed, only the tool and cut‐off used for depression have been validated against psychometric measures. However, all the tools were pretested with rural Zimbabwean women to ensure face and content validity of the questions and context relevance of the response options.

In this study of 4,025 women in rural Zimbabwe, maternal decision‐making autonomy, gender norm attitudes and social support during pregnancy significantly predicted attained linear growth of their children 2 years later, when the child was 18 months of age. These findings suggest that achieving global child health goals will require improvements in the empowerment of women within their societies. Interventions to provide social support (i.e. regular and reliable interaction with caring, trusted people) may be ‘lower hanging fruit’, but longer term interventions are underway to address power asymmetries and achieve gender equity.

## AUTHOR CONTRIBUTIONS

RJS, GHP, KLD, MAC, MNNM and CRM. conceptualized the study. MNNM, RN, AJP, JHH and CRM performed the research. JT, RN, JHH and CRM designed the methodology. JT, RM, RN, LHM and CRM analysed the data. JT, CRM and JHH drafted the paper; all authors critically reviewed and edited the final version.

## CONFLICTS OF INTEREST

The authors declare that they have no conflicts of interest.

## Supporting information


**Table S1.** Baseline maternal factors and infant factors at birth of study participants included in the analyses of maternal capabilities and child linear growth compared to participants who were not included in the analyses due to missing baseline maternal capability data or infant length‐for‐age‐Z score at 18 months.
Table S2. Association of maternal capabilities assessed during pregnancy with birth weight and low birth weight
Table S3. Covariates retained in final adjusted models for length for age Z score, stunting, birth weight, and low birth weight.
Table S4A: Association of maternal capabilities assessed during pregnancy with child length‐for‐age z score and stunting at 1 and 3 months of age
Table S4B: Association of maternal capabilities assessed during pregnancy with child length‐for‐age z score and stunting at 6 and 12 months of age

## References

[mcn13122-bib-0001] Aslam, M. , & Kingdon, G. (2012). Can education be a path to gender equality in the labour market? An update on Pakistan. Comparative Education, 48(2), 211–229.

[mcn13122-bib-0002] Avan, B. , Richter, L. M. , Ramchandani, P. G. , Norris, S. A. , & Stein, A. (2010). Maternal postnatal depression and children's growth and behaviour during the early years of life: Exploring the interaction between physical and mental health. Archives of Disease in Childhood, 95(9), 690–695. 10.1136/adc.2009.164848 20660522

[mcn13122-bib-0003] Bandura, A. (1977). Self‐efficacy: Toward a unifying theory of behavioral change. Psychological Review, 84, 191–215. 10.1037//0033-295x.84.2.191 847061

[mcn13122-bib-0004] Becker, M. H. (1974). The health belief model and personal health behavior. Health Education Monographs, 2, 324–473.

[mcn13122-bib-0005] Bronfenbrenner, U. (1986). Ecology of the family as a context for human development: Research perspectives. Developmental Psychology, 22(6), 723.

[mcn13122-bib-0006] Carlson, G. J. , Kordas, K. , & Murray‐Kolb, L. E. (2015). Associations between women's autonomy and child nutritional status: A review of the literature. Maternal & Child Nutrition, 11(4), 452–482. 10.1111/mcn.12113 24521434 PMC6860340

[mcn13122-bib-0007] Chang, H. Y. , Keyes, K. M. , Lee, K.‐S. , Choi, I. A. , Kim, S. J. , Kim, K. W. , & Shin, Y.‐J. (2014). Prenatal maternal depression is associated with low birth weight through shorter gestational age in term infants in Korea. Early Human Development, 90(1), 15–20. 10.1016/j.earlhumdev.2013.11.006 24331828 PMC5365071

[mcn13122-bib-0008] Chasekwa, B. , Maluccio, J. A. , Ntozini, R. , Moulton, L. H. , Wu, F. , Smith, L. E. , … Martin, S. L. (2018). Measuring wealth in rural communities: Lessons from the Sanitation, Hygiene, Infant Nutrition Efficacy (SHINE) trial. PLoS ONE, 13(6), e0199393. 10.1371/journal.pone.0199393 29953495 PMC6023145

[mcn13122-bib-0009] Chibanda, D. , Mangezi, W. , Tshimanga, M. , Woelk, G. , Rusakaniko, P. , Stranix‐Chibanda, L. , … Shetty, A. K. (2010). Validation of the Edinburgh Postnatal Depression Scale among women in a high HIV prevalence area in urban Zimbabwe. Archives of Women's Mental Health, 13(3), 201–206. 10.1007/s00737-009-0073-6 19760051

[mcn13122-bib-0010] Cohen, S. , Mermelstein, R. , Kamarck, T. , & Hoberman, H. M. (1985). Measuring the functional components of social support. In Social support: Theory, research and applications (pp. 73–94). New York: Springer.

[mcn13122-bib-0011] Cox, J. , Holden, J. , & Sagovsky, R. (1987). Edinburgh Postnatal Depression Scale (EPDS). The British Journal of Psychiatry, 150, 782–786. 10.1192/bjp.150.6.782 3651732

[mcn13122-bib-0012] Cunningham, K. , Ploubidis, G. B. , Menon, P. , Ruel, M. , Kadiyala, S. , Uauy, R. , & Ferguson, E. (2015). Women's empowerment in agriculture and child nutritional status in rural Nepal. Public Health Nutrition, 18(17), 3134–3145. 10.1017/S1368980015000683 25797070 PMC10271315

[mcn13122-bib-0013] Desai, S. , & Jain, D. (1994). Maternal employment and changes in family dynamics: The social context of women's work in rural South India. Population and Development Review, 20, 115–136. 10.2307/2137632

[mcn13122-bib-0014] Dewey, K. G. , & Begum, K. (2011). Long‐term consequences of stunting in early life. Maternal & Child Nutrition, 7, 5–18. 21929633 10.1111/j.1740-8709.2011.00349.x PMC6860846

[mcn13122-bib-0015] Dixon‐Mueller, R. B. (2013). Rural women at work: Strategies for development in South Asia. London: Routledge.

[mcn13122-bib-0016] Dumka, L. E. , Stoerzinger, H. D. , Jackson, K. M. , & Roosa, M. W. (1996). Examination of the cross‐cultural and cross‐language equivalence of the parenting self‐agency measure. Family Relations, 45, 216–222. 10.2307/585293

[mcn13122-bib-0017] Engle, P. L. , Menon, P. , & Haddad, L. (1999). Care and nutrition: Concepts and measurement. World Development, 27, 1309–1337.

[mcn13122-bib-0018] Field, T. , Diego, M. , Dieter, J. , Hernandez‐Reif, M. , Schanberg, S. , Kuhn, C. , … Bendell, D. (2004). Prenatal depression effects on the fetus and the newborn. Infant Behavior and Development, 27(2), 216–229.

[mcn13122-bib-0019] Fishbein, M. (1979). A theory of reasoned action: some applications and implications. 7242751

[mcn13122-bib-0020] Gilmore, L. , & Cuskelly, M. (2009). Factor structure of the parenting sense of competence scale using a normative sample. Child: Care, Health and Development, 35(1), 48–55. 10.1111/j.1365-2214.2008.00867.x 18991983

[mcn13122-bib-0021] Haider, R. , Ashworth, A. , Kabir, I. , & Huttly, S. R. A. (2000). Effect of community‐based peer counsellors on exclusive breastfeeding practices in Dhaka, Bangladesh: A randomised controlled trial. The Lancet, 356, 1643–1647. 10.1016/s0140-6736(00)03159-7 11089824

[mcn13122-bib-0022] Hays, R. D. , Sherbourne, C. D. , & Mazel, R. M. (1993). The RAND 36‐item health survey 1.0. Health Economics, 2(3), 217–227. 10.1002/hec.4730020305 8275167

[mcn13122-bib-0023] Hoddinott, J. , Behrman, J. R. , Maluccio, J. A. , Melgar, P. , Quisumbing, A. R. , Ramirez‐Zea, M. , … Martorell, R. (2013). Adult consequences of growth failure in early childhood. The American Journal of Clinical Nutrition, 98, 1170–1178. 10.3945/ajcn.113.064584 24004889 PMC3798075

[mcn13122-bib-0024] Hossain, B. (2020). Maternal empowerment and child malnutrition in Bangladesh. Applied Economics, 52(14), 1566–1581.

[mcn13122-bib-0025] Humphrey, J. H. , Mbuya, M. N. N. , Ntozini, R. , Moulton, L. H. , Stoltzfus, R. J. , Tavengwa, N. V. , … Team, S. H. I. N. E. S. T . (2019). Independent and combined effects of improved water, sanitation, and hygiene, and improved complementary feeding, on child stunting and anaemia in rural Zimbabwe: A cluster‐randomised trial. The Lancet Global Health, 7(1), e132–e147. 10.1016/S2214-109X(18)30374-7 30554749 PMC6293965

[mcn13122-bib-0026] Jones, R. , Haardörfer, R. , Ramakrishnan, U. , Yount, K. M. , Miedema, S. , & Girard, A. W. (2019). Women's empowerment and child nutrition: The role of intrinsic agency. SSM‐Population Health, 9, 100475. 31993480 10.1016/j.ssmph.2019.100475 PMC6978483

[mcn13122-bib-0027] Matare, C. R. , Mbuya, M. N. N. , Dickin, K. L. , Constas, M. A. , Pelto, G. , Chasekwa, B. , … Stoltzfus, R. J. (2020). Maternal Capabilities are associated with child caregiving behaviors among women in rural Zimbabwe. Journal of Nutrition. 10.1093/jn/nxaa255 PMC7948208 33211881

[mcn13122-bib-0028] Matare, C. R. , Mbuya, M. N. , Pelto, G. , Dickin, K. L. , & Stoltzfus, R. J. (2015). Assessing maternal capabilities in the SHINE trial: Highlighting a hidden link in the causal pathway to child health. Clinical Infectious Diseases, 61(suppl_7), S745–S751. 26602303 10.1093/cid/civ851 PMC4657596

[mcn13122-bib-0029] Maxwell, D. , Watkins, B. , Wheeler, R. , & Collins, G. (2003). The Coping Strategy Index: A tool for rapid measurement of household food security and the impact of food aid programs in humanitarian emergencies. CARE and WFP*,* Nairobi.

[mcn13122-bib-0030] Mbuya, M. N. N. , Matare, C. R. , Tavengwa, N. V. , Chasekwa, B. , Ntozini, R. , Majo, F. D. , … Team, S. T . (2019). Early Initiation and exclusivity of breastfeeding in rural Zimbabwe: Impact of a breastfeeding intervention delivered by village health workers. Curr Dev Nutr, 3(4), nzy092. 10.1093/cdn/nzy092 30937421 PMC6438822

[mcn13122-bib-0031] Murray, C. J. , Vos, T. , Lozano, R. , Naghavi, M. , Flaxman, A. D. , Michaud, C. , … Abdalla, S. (2012). Disability‐adjusted life years (DALYs) for 291 diseases and injuries in 21 regions, 1990–2010: A systematic analysis for the Global Burden of Disease Study 2010. The Lancet, 380(9859), 2197–2223. 10.1016/S0140-6736(12)61689-4 23245608

[mcn13122-bib-0032] Nanda, G. (2011). Compendium of gender scales (p. 360). Washington, DC: FHI.

[mcn13122-bib-0033] Nasreen, H. E. , Kabir, Z. N. , Forsell, Y. , & Edhborg, M. (2010). Low birth weight in offspring of women with depressive and anxiety symptoms during pregnancy: Results from a population based study in Bangladesh. BMC Public Health, 10(1), 515. 20796269 10.1186/1471-2458-10-515 PMC2939645

[mcn13122-bib-0034] Nussbaum, M. C. (2001). Women and human development: The capabilities approach (Vol. 3). Cambridge, UK: Cambridge University Press.

[mcn13122-bib-0035] Perry, H. , Morrow, M. , Borger, S. , Weiss, J. , DeCoster, M. , Davis, T. , & Ernst, P. (2015). Care Groups I: An innovative community‐based strategy for improving maternal, neonatal, and child health in resource‐constrained settings. Global Health: Science and Practice, 3(3), 358–369. 10.9745/GHSP-D-15-00051 PMC4570011 26374798

[mcn13122-bib-0036] Poobalan, A. S. , Aucott, L. S. , Ross, L. , Smith, W. C. S. , Helms, P. J. , & Williams, J. H. (2007). Effects of treating postnatal depression on mother–infant interaction and child development: Systematic review. The British Journal of Psychiatry, 191(5), 378–386. 17978316 10.1192/bjp.bp.106.032789

[mcn13122-bib-0037] Pratley, P. (2016). Associations between quantitative measures of women's empowerment and access to care and health status for mothers and their children: A systematic review of evidence from the developing world. Social Science & Medicine, 169, 119–131. 10.1016/j.socscimed.2016.08.001 27716549

[mcn13122-bib-0038] Prendergast, A. J. , Chasekwa, B. , Evans, C. , Mutasa, K. , Mbuya, M. N. N. , Stoltzfus, R. J. , … Humphrey, J. H. (2019). Independent and combined effects of improved water, sanitation, and hygiene, and improved complementary feeding, on stunting and anaemia among HIV‐exposed children in rural Zimbabwe: A cluster‐randomised controlled trial. The Lancet Child & Adolescent Health, 3(2), 77–90. 10.1016/s2352-4642(18)30340-7 30573417 PMC6472652

[mcn13122-bib-0039] Rahman, A. , Bunn, J. , Lovel, H. , & Creed, F. (2007). Association between antenatal depression and low birthweight in a developing country. Acta Psychiatrica Scandinavica, 115(6), 481–486. 10.1111/j.1600-0447.2006.00950.x 17498160 PMC1974771

[mcn13122-bib-0040] Saleem, A. F. , Mahmud, S. , Baig‐Ansari, N. , & Zaidi, A. K. (2014). Impact of maternal education about complementary feeding on their infants' nutritional outcomes in low‐and middle‐income households: A community‐based randomized interventional study in Karachi, Pakistan. Journal of Health, Population, and Nutrition, 32(4), 623. PMC4438693 25895196

[mcn13122-bib-0041] Santoso, M. V. , Kerr, R. B. , Hoddinott, J. , Garigipati, P. , Olmos, S. , & Young, S. L. (2019). Role of women's empowerment in child nutrition outcomes: A systematic review. Advances in Nutrition, 10(6), 1138–1151. 10.1093/advances/nmz056 31298299 PMC6855975

[mcn13122-bib-0042] Sethuraman, K. , Lansdown, R. , & Sullivan, K. (2006). Women's empowerment and domestic violence: The role of sociocultural determinants in maternal and child undernutrition in tribal and rural communities in South India. Food and Nutrition Bulletin, 27(2), 128–143. 10.1177/156482650602700204 16786979

[mcn13122-bib-0043] Shafiq, A. , Hussain, A. , Asif, M. , Hwang, J. , Jameel, A. , & Kanwel, S. (2019). The effect of “women's empowerment” on child nutritional status in Pakistan. International Journal of Environmental Research and Public Health, 16(22), 4499. 10.3390/ijerph16224499 PMC6888433 31739650

[mcn13122-bib-0044] Sherbourne, C. D. , & Stewart, A. L. (1991). The MOS social support survey. Social Science & Medicine, 32(6), 705–714. 10.1016/0277-9536(91)90150-b 2035047

[mcn13122-bib-0045] SHINE Trial Team . (2015). The Sanitation Hygiene Infant Nutrition Efficacy (SHINE) trial: Rationale, design, and methods. CID, 61(suppl 7), S685–S702. 10.1093/cid/civ844 PMC4657589 26602296

[mcn13122-bib-0046] Shrimpton, R. , Victora, C. G. , De Onis, M. , Lima, R. C. , Blossner, M. , & Clugston, G. (2001). Worldwide timing of growth faltering: Implications for nutritional interventions. Pediatrics, 107(5), 1–7. 11331725 10.1542/peds.107.5.e75

[mcn13122-bib-0047] Shroff, M. , Griffiths, P. , Adair, L. , Suchindran, C. , & Bentley, M. (2009). Maternal autonomy is inversely related to child stunting in Andhra Pradesh, India. Maternal & Child Nutrition, 5(1), 64–74. 10.1111/j.1740-8709.2008.00161.x 19161545 PMC3811039

[mcn13122-bib-0048] Singh, K. , Haney, E. , & Olorunsaiye, C. (2013). Maternal autonomy and attitudes towards gender norms: Associations with childhood immunization in Nigeria. Maternal and Child Health Journal, 17(5), 837–841. 10.1007/s10995-012-1060-5 22696106 PMC3966061

[mcn13122-bib-0049] Surkan, P. J. , Kennedy, C. E. , Hurley, K. M. , & Black, M. M. (2011). Maternal depression and early childhood growth in developing countries: Systematic review and meta‐analysis. Bulletin of the World Health Organization, 89, 607–615. 10.2471/BLT.11.088187 PMC3150769 21836759

[mcn13122-bib-0050] Tuthill, E. L. , McGrath, J. M. , Graber, M. , Cusson, R. M. , & Young, S. L. (2016). Breastfeeding self‐efficacy: A critical review of available instruments. Journal of Human Lactation, 32(1), 35–45. 10.1177/0890334415599533 26319113 PMC4882127

[mcn13122-bib-0051] Undlien, M. , Viervoll, H.‐A. , & Rostad, B. (2016). Effect of Mother Support Groups on nutritional status in children under two years of age in Laisamis village, Kenya. African Health Sciences, 16(4), 904–909. 10.4314/ahs.v16i4.4 28479880 PMC5398434

[mcn13122-bib-0052] UNICEF . (1990). Strategy for improved nutrition of children and women in developing countries. 10.1007/BF02810402 1937618

[mcn13122-bib-0053] UNICEF , WHO , & World Bank . (2017). Levels and trends in child malnutrition. Joint child malnutrition estimates (p. 2017). New York, NY: United Nations International Children's Fund; Geneva:WHO:Washington, DC:World Bank.

[mcn13122-bib-0054] Upvall, M. J. , Sochael, S. , & Gonsalves, A. (2002). Behind the mud walls: The role and practice of lady health visitors in Pakistan. Health Care for Women International, 23(5), 432–441. 10.1080/073993302760190038 12171694

[mcn13122-bib-0055] Wado, Y. D. , Afework, M. F. , & Hindin, M. J. (2014). Effects of maternal pregnancy intention, depressive symptoms and social support on risk of low birth weight: A prospective study from southwestern Ethiopia. PLoS ONE, 9(5), e96304. 10.1371/journal.pone.0096304 24848269 PMC4029816

[mcn13122-bib-0056] World Health Organization . (2012). Depression: A global public health concern. Geneva, Switzerland: WHO.

[mcn13122-bib-0057] Yount, K. M. (2004). Maternal resources, proximity of services, and curative care of boys and girls in Minya, Egypt 1995–97. Population Studies, 58(3), 345–355. 10.1080/0032472042000272384 15513288

