# Supplementary material for: Maternal caregiving capabilities are associated with child linear growth in rural Zimbabwe
Source: Matern Child Nutr. 2020 Dec 21;17(2):e13122. doi: 10.1111/mcn.13122 (PMC7988870; doi:10.1111/mcn.13122)
Supplement: Supplementary file 1 — Table S1. Baseline maternal factors and infant factors at birth of study participants included in the analyses of maternal capabilities and child linear growth compared to participants who were not included in the analyses due to missing baseline maternal capability data or infant length‐for‐age‐Z score at 18 months. Table S2. Association of maternal capabilities assessed during pregnancy with birth weight and low birth weight Table S3. Covariates retained in final adjusted models for length for age Z score, stunting, birth weight, and low birth weight. Table S4A: Association of maternal capabilities assessed during pregnancy with child length‐for‐age z score and stunting at 1 and 3 months of age Table S4B: Association of maternal capabilities assessed during pregnancy with child length‐for‐age z score and stunting at 6 and 12 months of age [file MCN-17-e13122-s001.docx]

**Supplementary Online Material**

**Joice Tome, et al: Maternal care-giving capabilities assessed during pregnancy predict linear growth in their children: a longitudinal study in rural Zimbabwe**

**Supplementary Table 1.**  Baseline maternal factors and infant factors at birth of study participants included in the analyses of maternal capabilities and child linear growth compared to participants who were not included in the analyses due to missing baseline maternal capability data or infant length-for-age-Z score at 18 months.

Supplementary Table 2. Association of maternal capabilities assessed during pregnancy with birth weight and low birth weight

Supplementary Table 3. Covariates retained in final adjusted models for length for age Z score, stunting, birth weight, and low birth weight.

Supplementary Table 4A: Association of maternal capabilities assessed during pregnancy with child length-for-age z score and stunting at 1 and 3 months of age

Supplementary Table 4B: Association of maternal capabilities assessed during pregnancy with child length-for-age z score and stunting at 6 and 12 months of age

Supplementary Table 1. Baseline maternal factors and infant factors at birth of study participants included in the analyses of maternal capabilities and child linear growth compared to participants who were not included in the analyses due to missing baseline maternal capability data or infant length-for-age-Z score at 18 months. ^†^

| ***Mother's characteristics at baseline*** | **Included in analyses**  **N=4025** | **Excluded in analyses**  **N = 394**^‡^ | **P** ^§^ **value** | |
| --- | --- | --- | --- | --- |
| Mean age( SD), y | 26.3 ( 6.6) | 24.8 (6.1) | 0.192 |  |
| Mean height (SD), cm | 160.2 ( 5.9) | 160.3 (6.5) | 0.642 |  |
| Mean mid-upper-arm circumference (SD), cm | 26.4 ( 3.0) | 26.3 (3.5) | 0.699 |  |
| Mean years of schooling completed (SD) | 9.5 ( 1.8) | 9.5 (3.0) | 0.821 |  |
| Married, n/total n (%) | 3660/3828 (95.6%) | 250/281 (89.0%) | <0.001 |  |
| Employed, n/total n (%) | 348/4007 (8.7%) | 1/17 (5.9%) | 0.686 |  |
| Religion, n/total n(%) |  |  |  |  |
| Apostolic | 1797/3861 (46.5%) | 142/280 (50.7%) | 0.339 |  |
| Other Christians | 1737/3861 (45.0%) | 119/280 (42.5%) |  |  |
| Other religions | 327/3861 (8.5%) | 19/280 (6.8%) |  |  |
| HIV status, n/total n(%) |  |  |  |  |
| Positive | 642/4025 (16.0%) | 16/394 (4.1%) | <0.001 |  |
| Negative | 3374/4025 (83.8%) | 280/394 (71.1%) |  |  |
| Unknown | 9/4025 (0.2%) | 98/394 (24.9%) |  |  |
|  |  |  |  |  |
| ***Infant characteristics at birth*** | **Infants included in analyses N=4073** | **Infants excluded in analyses N=394**^‡^ |  |  |
|  |  |  |  |  |
| Mean LAZ (SD) | -1.6 (1.1) | -1.5 (1.1) | 0.360 |  |
| Stunting, n/total n(%) | 1361/4073 (33.4%) | 110/340 (32.4%) | 0.729 |  |
| Female sex, n/total n(%) | 2031/4073 (49.9%) | 201/388 (51.8%) | 0.450 |  |
| Mean birth weight (SD), *kg* | 3.1 (0.5) [3740] | 3.1 (0.5) | 0.177 |  |
| Birth weight < 2500g, n/total n(%) | 325/3740 (8.7%) | 36/327 (11.0%) | 0.133 |  |
| Mean gestational age(SD),weeks | 38.6 (3.7) | 37.7 (5.4) | 0.002 |  |
| Multiple birth, n/total n(%) | 108/4073 (2.7%) | 7/394 (1.8%) | 0.290 |  |

^†^Fewer than 5% of data are missing unless N is stated.

^‡^378 mothers missing maternal capabilities + 16 infants missing LAZ

^‡^378 mothers missing maternal capabilities + 16 infants missing LAZ

^§^ p-values based on xtgee, multinomial, ordinal regression models with robust variance estimation, and Somers' D for medians.

Supplementary Table 2: Association of maternal capabilities assessed during pregnancy with birth weight and low birth weight

| **Maternal Capability** |  | **Birth weight** | | | | |  | **Low birth weight (< 2500 g)** | | | | |
| --- | --- | --- | --- | --- | --- | --- | --- | --- | --- | --- | --- | --- |
|  |  | **Unadjusted**  **β (95% C.I)** ^†^ | | | **Adjusted**  **β (95% C.I)** ^‡^ | |  | **Unadjusted**  **OR (95% C.I) ^†^** | | | **Adjusted**  **OR (95% C.I)** ^‡^ | |
| **Decision making autonomy** |  | 0.01(-0.003;0.02) 0.15 | | | -0.001(-0.01;0.01) 0.88 | |  | 0.96(0.87;1.05)  0.37 | | | 1.02(0.92;1.14)  0.65 | |
|  |  |  | | |  | |  |  | | |  | |
| **Gender norm attitudes** |  | 0.03(0.04; 0.06) 0.05 | | | 0.01(-0.02;0.04) 0.52 | |  | 0.88(0.70;1.12)  0.32 | | | 1.00(0.78;1.28)  0.99 | |
|  |  |  | | |  | |  |  | | |  | |
| **Depression** |  | -0.01 (-0.07, 0.05) 0.84 | | | 0.02 (-0.03;0.08) 0.35 | |  | 0.78 (0.47, 1.27) 0.31 | | | 0.65 (0.39;1.09) 0.10 | |
|  |  |  | | |  | |  |  | | |  | |
| **Mothering self- efficacy** |  | 0.07 (0.04;0.10) <0.01 | | | 0.03 (0.001;0.06) 0.05 | |  | 0.82 (0.62;1.09) 0.17 | | | 0.91 (0.66;1.27) 0.59 | |
|  |  |  | | |  | |  |  | | |  | |
| **Perceived health status** |  | -0.01 (-0.03;0.01) 0.19 | | | -0.01 (-0.02;0.01) 0.38 | |  | 1.05 (0.92;1.19) 0.46 | | | 1.03 (0.91;1.18) 0.64 | |
|  |  |  | | |  | |  |  | | |  | |
| **Perceived social support** |  | 0.01 (-0.02;0.04) 0.47 | | | 0.001 (-0.03;0.03) 0.95 | |  | 0.89 (0.73;1.09) 0.26 | | | 0.94 (0.76;1.16) 0.56 | |
|  |  |  | | |  | |  |  | | |  | |
| **Perceived time stress** |  | 0.01 (-0.01;0.03)  0.35 | | | 0.003 (-0.02;0.02) 0.78 | |  | 0.91 (0.78;1.07) 0.25 | | | 0.93 (0.78;1.11) 0.42 | |
|  | | |  |  | |  | | |  |  | |  |

^†^ Unadjusted models- adjusted for cluster and intervention arms to account for within-cluster correlation and controlling for the intervention effect.

^‡^ Adjusted models- adjusted for cluster, intervention arms and other baseline covariates which were associated with the exposure and outcome at p<0.2. Baseline variables tested: age of mother at birth, mother’s height, mother’s body mass index, mother’s mid upper arm circumference, mother’s level of education, receipt of WASH intervention, receipt of IYCF intervention, child birth order, child sex, single or multiple birth, birth interval, time to water source, improved latrine, improved water source, household wealth index, presence of functioning hand washing station with water and soap, coping strategy index, gender of household head, feces observed in yard, number of children under-fives, household size, treated water, father’s level of education, mother’s employment outside home, household dietary diversity, mother’s HIV status at enrolment. See Supplementary Table 3 for lists of covariates retained in each final adjusted model.

Supplementary Table 3. Covariates retained in final adjusted models for length for age Z score, stunting, birth weight, and low birth weight.

| **Maternal capabilities** | **LAZ adjusted** | **Stunting adjusted** | **Birth weight adjusted** | **LBW adjusted** |
| --- | --- | --- | --- | --- |
| **Decision making autonomy** | age of mother at birth, mother’s height, mother’s mid upper arm circumference, receipt of IYCF intervention, child sex, single or multiple birth, mother’s level of education, improved water source, household wealth index, presence of functioning hand washing station with water and soap, feces observed in yard, mother’s HIV status at enrolment. | age of mother at birth, mother’s height, mother’s mid upper arm circumference, receipt of IYCF intervention, child sex, single or multiple birth, household wealth index, presence of functioning hand washing station with water and soap, gender of household head, number of children under-fives, mother’s HIV status at enrolment. | age of mother at birth, mother’s height, mother’s mid upper arm circumference, child birth order, child sex, single or multiple birth, improved water source, household size, treated water, number of children under-fives, mother’s HIV status at enrolment | age of mother at birth, mother’s height, mother’s mid upper arm circumference, child sex, single or multiple birth, improved water source, coping strategy index, number of children under-fives, household size, mother’s HIV status at enrolment |
| **Gender norm attitudes** | age of mother at birth, mother’s height, mother’s mid upper arm circumference, mother’s level of education, receipt of IYCF intervention, child sex, single or multiple birth, improved water source, household wealth index, presence of functioning hand washing station with water and soap, feces observed in yard, number of children under-fives, mother’s HIV status at enrolment. | age of mother at birth, mother’s height, mother’s mid upper arm circumference, receipt of IYCF intervention, child sex, single or multiple birth, household wealth index, presence of functioning hand washing station with water and soap, gender of household head, number of children under-fives, mother’s HIV status at enrolment | age of mother at birth, mother’s height, mother’s mid upper arm circumference, child birth order, child sex, single or multiple birth, receipt of WASH intervention , improved water source, number of children under-fives, household size, mother’s HIV status at enrolment | age of mother at birth, mother’s height, mother’s mid upper arm circumference, child sex, single or multiple birth, presence of functioning hand washing station with water and soap, coping strategy index, gender of household head, number of children under-fives, mother’s HIV status at enrolment |
| ***IYCF group*** | mother’s height, mother’s mid upper arm circumference, receipt of WASH intervention, child sex, single or multiple birth, household wealth index, presence of functioning hand washing station with water and soap, feces observed in yard, mother’s HIV status at enrolment. | mother’s height, mother’s mid upper arm circumference, child sex, single or multiple birth, household wealth index, feces observed in yard, treated water, mother’s HIV status at enrolment |  |  |
| ***Non-IYCF group*** | mother’s height, mother’s mid upper arm circumference, child sex, single or multiple birth, improved water source, household wealth index, number of children under-fives household dietary diversity, mother’s HIV status at enrolment. | mother’s height, mother’s mid upper arm circumference, mother’s level of education, child birth order, child sex, single or multiple birth, household wealth index, gender of household head, number of children under-fives, household dietary diversity, mother’s HIV status at enrolment |  |  |
| **Depression** | mother’s height, mother’s mid upper arm circumference, child sex, single or multiple birth, presence of functioning hand washing station with water and soap, feces observed in yard, number of children under-fives, household dietary diversity, mother’s HIV status at enrolment. | age of mother at birth, mother’s height, mother’s mid upper arm circumference, , receipt of IYCF intervention, child sex, single or multiple birth, household wealth index, presence of functioning hand washing station with water and soap, gender of household head, number of children under-fives, household dietary diversity, mother’s HIV status at enrolment | mother’s height, mother’s mid upper arm circumference, receipt of WASH intervention, child birth order, child sex, single or multiple birth, improved water source, number of children within a household, number of children under-fives, household size, treated water, mother’s HIV status at enrolment | mother’s height, mother’s mid upper arm circumference, mother’s level of education, single or multiple birth, gender of household head, number of children under-fives, mother’s HIV status at enrolment, number of children within a household |
| **IYCF group** | mother’s height, mother’s mid upper arm circumference, child sex, single or multiple birth, household wealth index, number of children under-fives, mother’s HIV status at enrolment. | mother’s height, mother’s mid upper arm circumference, child sex, single or multiple birth, household wealth index, mother’s HIV status at enrolment |  |  |
| **Non-IYCF group** | mother’s height, mother’s mid upper arm circumference, child sex, single or multiple birth, improved latrine, household wealth index, gender of household head, number of children under-fives, household size, household dietary diversity, mother’s HIV status at enrolment. | age of mother at birth, mother’s height, mother’s mid upper arm circumference, mother’s level of education, child birth order, child sex, single or multiple birth, household wealth index, presence of functioning hand washing station with water and soap, gender of household head, household dietary diversity, mother’s HIV status at enrolment |  |  |
| **Mothering self_efficacy** | mother’s height, mother’s mid upper arm circumference, receipt of IYCF intervention, child sex, single or multiple birth, feces observed in yard, number of children under-fives, household dietary diversity, mother’s HIV status at enrolment. | age of mother at birth, mother’s height, mother’s mid upper arm circumference, receipt of IYCF intervention, child sex, household wealth index, single or multiple birth, number of children under-fives, mother’s HIV status at enrolment | age of mother at birth, mother’s height, mother’s mid upper arm circumference, receipt of WASH intervention, child birth order, child sex, single or multiple birth, improved water source, number of children under-fives, household size, treated water, mother’s HIV status at enrolment, number of children within a household | age of mother at birth, mother’s height, mother’s mid upper arm circumference, child sex, single or multiple birth, receipt of WASH intervention , coping strategy index, gender of household head, number of children under-fives, mother’s HIV status at enrolment |
| **Perceived health status** | age of mother at birth, mother’s height, mother’s mid upper arm circumference, receipt of IYCF intervention, child sex, single or multiple birth, improved latrine, improved water source, number of children under-fives, mother’s HIV status at enrolment. | age of mother at birth, mother’s height, mother’s mid upper arm circumference, receipt of IYCF intervention, child sex, single or multiple birth, improved latrine, improved water source, household wealth index, gender of household head, number of children under-fives, mother’s HIV status at enrolment | age of mother at birth, mother’s height, mother’s mid upper arm circumference, mother’s level of education, child birth order, child sex, single or multiple birth, improved water source, number of children under-fives, household size, treated water, mother’s HIV status at enrolment, number of children within a household | age of mother at birth, mother’s height, mother’s mid upper arm circumference, receipt of WASH intervention, mother’s level of education, single or multiple birth, gender of household head, number of children under-fives, mother’s HIV status at enrolment |
| **Perceived social support** | mother’s height, mother’s mid upper arm circumference, mother’s level of education, receipt of IYCF intervention, child sex, single or multiple birth, presence of functioning hand washing station with water and soap, number of children under-fives. | mother’s height, mother’s mid upper arm circumference receipt of IYCF intervention, child sex, single or multiple birth, household wealth index, presence of functioning hand washing station with water and soap, feces observed in yard, number of children under-fives, mother’s HIV status at enrolment | age of mother at birth, mother’s height, mother’s mid upper arm circumference, receipt of WASH intervention, child sex, single or multiple birth, child birth order, improved water source, number of children under-fives, household size, treated water, mother’s HIV status at enrolment | mother’s height, mother’s mid upper arm circumference, receipt of WASH, child sex, single or multiple birth, coping strategy index, number of children under-fives, mother’s HIV status at enrolment |
| **Perceived time stress** | mother’s height, mother’s mid upper arm circumference, receipt of IYCF intervention, child sex, single or multiple birth, presence of functioning hand washing station with water and soap, feces observed in yard, number of children under-fives, household dietary diversity, mother’s HIV status at enrolment. | age of mother at birth, mother’s height, mother’s mid upper arm circumference, receipt of IYCF intervention, child sex, single or multiple birth, household wealth index, presence of functioning hand washing station with water and soap, feces observed in yard, number of children under-fives, household dietary diversity, mother’s HIV status at enrolment | age of mother at birth, mother’s height, mother’s mid upper arm circumference, receipt of WASH intervention, child birth order, child sex, single or multiple birth, improved water source, number of children under-fives, household size, treated water, mother’s HIV status at enrolment | age of mother at birth, mother’s height, mother’s mid upper arm circumference, receipt of WASH intervention, child sex, single or multiple birth, presence of functioning hand washing station with water and soap, coping strategy index, number of children under-fives, mother’s HIV status at enrolment |

Supplementary Table 4A: Association of maternal capabilities assessed during pregnancy with child length-for-age z score and stunting at 1 and 3 months of age

| **Maternal Capability** |  | **Length-for-Age Z score at 1 mo** | |  | **Stunted (LAZ<−2.0) at 1 mo** | |  | **Length-for-Age Z score at 3 mo** | |  | **Stunted (LAZ<−2.0) at 3 mo** | |
| --- | --- | --- | --- | --- | --- | --- | --- | --- | --- | --- | --- | --- |
|  |  | **Unadjusted**  **β (95% C.I)** ^†^ | **Adjusted**  **β (95% C.I)** ^‡^ |  | **Unadjusted**  **OR (95% C.I)** ^†^ | **Adjusted**  **OR (95% C.I)** ^‡^ |  | **Unadjusted**  **β (95% C.I)** ^†^ | **Adjusted**  **β (95% C.I)** ^‡^ |  | **Unadjusted**  **OR (95% C.I)** ^†^ | **Adjusted**  **OR (95% C.I)** ^‡^ |
| **Decision making autonomy** |  | 0.02 (-0.02; 0.07)  0.31 | 0.02 (-0.03;0.06)  0.52 |  | 1.00 (0.92; 1.09)  0.  97 | 1.02 (0.93; 1.12)  0.68 |  | 0.05 (0.01; 0.09)  0.02 | 0.02 (-0.02;0.06)  0.34 |  | 0.91 (0.84; 0.99)  0.03 | 0.94 (0.86; 1.03)  0.20 |
|  |  |  |  |  |  |  |  |  |  |  |  |  |
| **Gender norm attitudes**^§^ |  | 0.03 (-0.06; 0.12)  0.52 | 0.001(-0.09;0.09)  0.98 |  | 0.92 (0.75; 1.14)  0.47 | 0.99 (0.79; 1.23)  0.91 |  | 0.01 (-0.09;0.10)  0.88 | -0.02(-0.13;0.08)  0.68 |  | 0.99 (0.80; 1.22)  0.90 | 1.04 (0.83; 1.29)  0.75 |
| **Depression** |  | -0.07(-0.28;0.14)  0.50 | 0.05 (-0.17;0.27)  0.67 |  | 1.12 (0.76; 1.63)  0.57 | 0.84 (0.55; 1.28)  0.42 |  | -0.15(-0.34;0.04)  0.13 | -0.05(-0.22;0.13)  0.61 |  | 0.98 (0.64; 1.54)  0.93 | 0.83 (0.53; 1.30)  0.42 |
| **Perceived social support** |  | 0.05 (-0.04;0.14)  0.26 | 0.01 (-0.09;0.10)  0.88 |  | 1.02 (0.84; 1.24)  0.85 | 1.08 (0.87; 1.33)  0.51 |  | 0.10 (0.01; 0.20)  0.04 | 0.06(-0.02;0.15)  0.15 |  | 0.86 (0.70; 1.06)  0.16 | 0.92 (0.75; 1.13)  0.44 |
| **Mothering self- efficacy** |  | 0.04 (-0.10;0.18)  0.54 | -0.001  (-0.15;0.14)  0.99 |  | 0.98 (0.72; 1.34)  0.92 | 1.05 (0.78; 1.42)  0.73 |  | -0.10(-0.22;0.03)  0.12 | -0.14(-0.28;-0.01)  0.03 |  | 1.10 (0.85; 1.42)  0.47 | 1.13 (0.87, 1.47)  0.36 |
|  |  |  |  |  |  |  |  |  |  |  |  |  |
| **Perceived health status** |  | 0.005(0.04;0.05)  0.84 | 0.01 (-0.03;0.06)  0.53 |  | 0.88 (0.79; 0.98)  0.02 | 0.86 (0.77; 0.97)  0.02 |  | 0.04 (-0.22;0.09)  0.19 | 0.04(-0.01;0.09)  0.12 |  | 0.99 (0.87; 1.13)  0.87 | 0.99 (0.87; 1.13)  0.91 |
|  |  |  |  |  |  |  |  |  |  |  |  |  |
|  |  |  |  |  |  |  |  |  |  |  |  |  |
| **Perceived time stress** |  | -0.01(-0.08;0.05)  0.70 | -0.04(-0.11;0.03)  0.29 |  | 1.10 (0.94; 1.29)  0.23 | 1.14 (0.95; 1.37)  0.17 |  | -0.03(-0.10;0.04)  0.45 | -0.05(-0.13;0.02)  0.15 |  | 0.94 (0.82; 1.08)  0.41 | 0.93 (0.80; 1.08)  0.32 |

^†^ Unadjusted models- adjusted for cluster and intervention arms to account for within-cluster correlation and controlling for the intervention effect.

^‡^ Adjusted models- adjusted for cluster, intervention arms and other baseline covariates which were associated with the exposure and outcome at p<0.2. Baseline variables tested: age of mother at birth, mother’s height, mother’s body mass index, mother’s mid upper arm circumference, mother’s level of education, receipt of WASH intervention, receipt of IYCF intervention, child birth order, child sex, single or multiple birth, birth interval, time to water source, improved latrine, improved water source, household wealth index, presence of functioning hand washing station with water and soap, coping strategy index, gender of household head, feces observed in yard, number of children under-fives, household size, treated water, father’s level of education, mother’s employment outside home, household dietary diversity, mother’s HIV status at enrolment. See Supplementary Table 3 for lists of covariates retained in each final adjusted model.

Supplementary Table 4B: Association of maternal capabilities assessed during pregnancy with child length-for-age z score and stunting at 6 and 12 months of age

| **Maternal Capability** |  | **Length-for-Age Z score at 6 mo** | |  | **Stunted (LAZ<−2.0) at 6 mo** | |  | **Length-for-Age Z score at 12 mo** | |  | **Stunted (LAZ<−2.0) at 12 mo** | |
| --- | --- | --- | --- | --- | --- | --- | --- | --- | --- | --- | --- | --- |
|  |  | **Unadjusted**  **β (95% C.I)** ^†^ | **Adjusted**  **β (95% C.I)** ^‡^ |  | **Unadjusted**  **OR (95% C.I)** ^†^ | **Adjusted**  **OR (95% C.I)** ^‡^ |  | **Unadjusted**  **β (95% C.I)** ^†^ | **Adjusted**  **β (95% C.I)** ^‡^ |  | **Unadjusted**  **OR (95% C.I)** ^†^ | **Adjusted**  **OR (95% C.I)** ^‡^ |
| **Decision making autonomy** |  | 0.04 (0.004;0.08)  0.03 | 0.02(-0.01;0.06)  0.19 |  | 0.94(0.87;1.02)  0.15 | 0.96(0.88;1.04)  0.31 |  | 0.04(0.01;0.07)  0.01 | 0.02(-0.004;0.05)  0.10 |  | 0.92(0.87;0.98)  0.01 | 0.94(0.88;0.10)  0.04 |
|  |  |  |  |  |  |  |  |  |  |  |  |  |
| **Gender norm attitudes** |  | 0.03(-0.04;0.11)  0.40 | 0.01(-0.06;0.09)  0.74 |  | 1.01(0.84;1.21)  0.89 | 1.06(0.86;1.31)  0.57 |  | 0.07(-0.001;0.14)  0.05 | 0.03(-0.04;0.10)  0.40 |  | 0.91(0.80;1.04)  0.18 | 0.98(0.84;1.14)  0.79 |
|  |  |  |  |  |  |  |  |  |  |  |  |  |
| **Depression** |  | -0.04(-0.22;0.14)  0.65 | -0.02(-0.19;0.14)  0.79 |  | 1.02(0.72;1.46)  0.90 | 0.82(0.53;1.28)  0.39 |  | -0.14(0.30;0.01)  0.07 | -0.09(-0.23;0.06)  0.24 |  | 1.23(0.92;1.65)  0.15 | 1.13(0.84;1.52)  0.43 |
| **Perceived social support** |  | 0.09(0.01;0.18)  0.04 | 0.07(-0.01;0.15)  0.08 |  | 0.83(0.71;0.98)  0.03 | 0.91(0.76;1.09)  0.31 |  | 0.12(0.05;0.19)  0.01 | 0.10(0.03;0.18)  0.01 |  | 0.85(0.72;1.00)  0.06 | 0.89(0.75;1.06)  0.21 |
| **Mothering self- efficacy** |  | -0.03(-0.15;0.08)  0.04 | -0.02(-0.13;0.09)  0.72 |  | 0.89(0.69;1.16)  0.40 | 0.91(0.68;1.22)  0.52 |  | 0.06(-0.14;0.15)  0.22 | 0.05(-0.03;0.14)  0.24 |  | 0.94(0.78;1.13)  0.50 | 0.94(0.77;1.14)  0.51 |
|  |  |  |  |  |  |  |  |  |  |  |  |  |
| **Perceived health status** |  | 0.01(-0.04;0.06)  0.81 | 0.01(-0.03;0.05)  0.82 |  | 0.97(0.88;1.07)  0.56 | 0.97(0.87;1.09)  0.62 |  | 0.04(0.001;0.09)  0.06 | 0.05(0.01;0.09)  0.02 |  | 0.93(0.86;1.02)  0.12 | 0.92(0.84;1.01)  0.07 |
| **Perceived time stress** |  | -0.05(-0.11;0.001)  0.06 | -0.05(-0.11;0.001)  0.06 |  | 1.03(0.91;1.17)  0.64 | 1.01(0.87;1.16)  0.94 |  | -0.06(-0.11;0.003)  0.04 | -0.06(-0.11;-0.01)  0.12 |  | 1.13(1.01;1.27)  0.03 | 1.17(1.03;1.31)  0.01 |

^†^ Unadjusted models- adjusted for cluster and intervention arms to account for within-cluster correlation and controlling for the intervention effect.

^‡^ Adjusted models- adjusted for cluster, intervention arms and other baseline covariates which were associated with the exposure and outcome at p<0.2. Baseline variables tested: age of mother at birth, mother’s height, mother’s body mass index, mother’s mid upper arm circumference, mother’s level of education, receipt of WASH intervention, receipt of IYCF intervention, child birth order, child sex, single or multiple birth, birth interval, time to water source, improved latrine, improved water source, household wealth index, presence of functioning hand washing station with water and soap, coping strategy index, gender of household head, feces observed in yard, number of children under-fives, household size, treated water, father’s level of education, mother’s employment outside home, household dietary diversity, mother’s HIV status at enrolment. See Supplementary Table 3 for lists of covariates retained in each final adjusted model.
